# Supplementary material for: Predicting visual function by interpreting a neuronal wiring diagram
Source: Nature. 2024 Oct 2;634(8032):113–23. doi: 10.1038/s41586-024-07953-5 (PMC11446822; doi:10.1038/s41586-024-07953-5)
Supplement: Supplementary file 6 — CRF and ERF predictions for individual TmY4 and TmY9 cells. Analogous to Supplementary Data 3, but for TmY target types. Shown are the top four monosynaptic pathways, the strongest pathway passing through each of the top ten intermediary types (ranking from Extended Data Fig. 7), and the trisynaptic pathway Tm1–TmY–Dm3–TmY (see the section entitled Prediction of spatial normalization). [file 41586_2024_7953_MOESM6_ESM.zip › DataS4/TmY9qΓèÑ/720575940629050128.pdf]

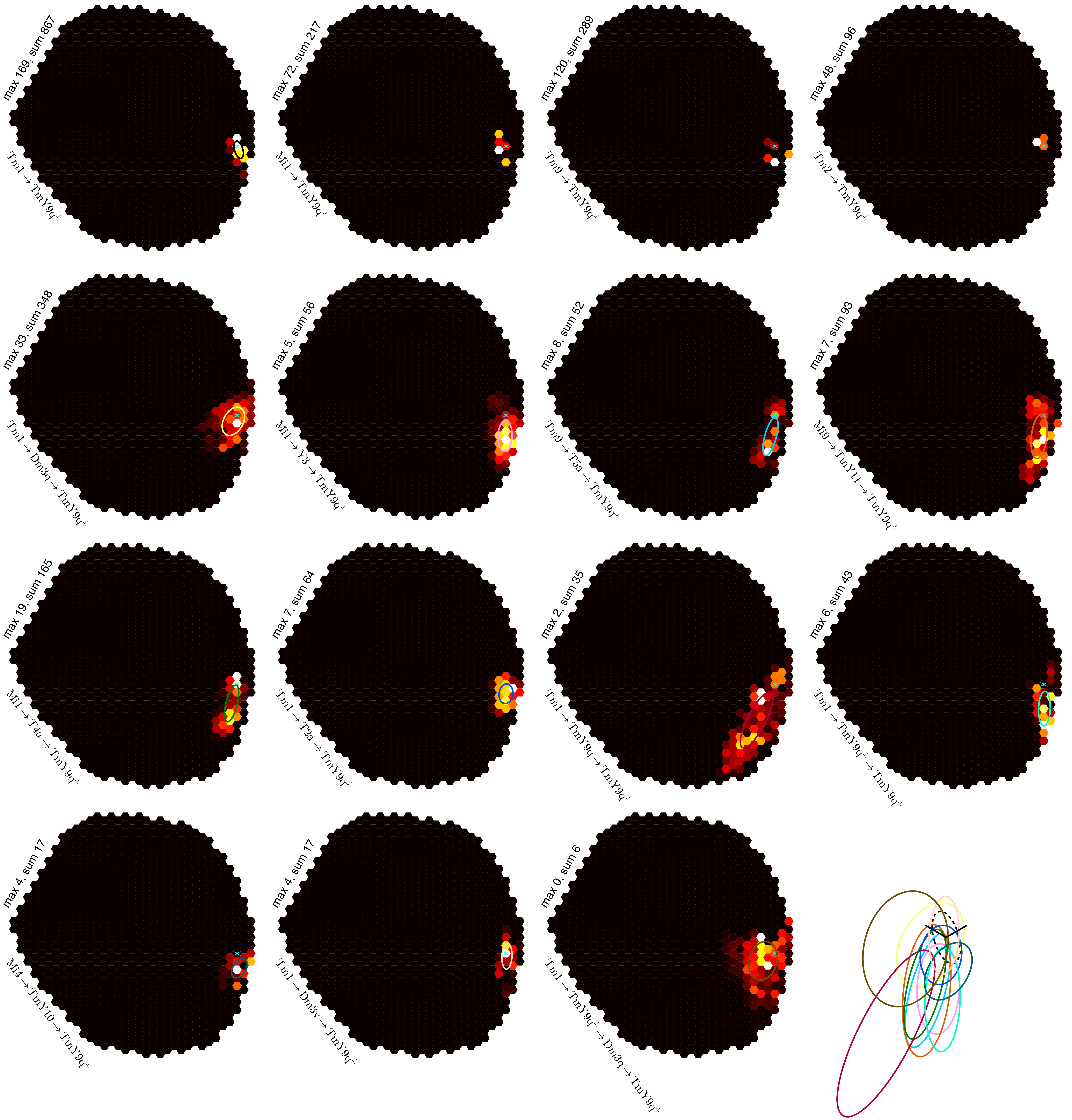
max 169, sum 867
Tm1 → TmY9q⊥
max 72, sum 217
Mi1 → TmY9q⊥
max 120, sum 289
Tm9 → TmY9q⊥
max 48, sum 96
Tm2 → TmY9q⊥
max 33, sum 348
Tm1 → Dm3q → TmY9q⊥
max 5, sum 56
Mi1 → Y3 → TmY9q⊥
max 8, sum 52
Tm9 → T5a → TmY9q⊥
max 7, sum 93
Mi9 → TmY11 → TmY9q⊥
max 19, sum 165
Mi1 → T4a → TmY9q⊥
max 7, sum 64
Tm1 → T2a → TmY9q⊥
max 2, sum 35
Tm1 → TmY9q → TmY9q⊥
max 6, sum 43
Tm1 → TmY9q⊥ → TmY9q⊥
max 4, sum 17
Mi4 → TmY10 → TmY9q⊥
max 4, sum 17
Tm1 → Dm3v → TmY9q⊥
max 0, sum 6
Tm1 → TmY9q⊥ → Dm3q → TmY9q⊥
